# Interpreting oral microbiome research in clinical dentistry: terminology, limitations and clinical implications

**DOI:** 10.1038/s41415-026-9896-z

**Published:** 2026-08-28

**Authors:** Kitty Guo, David Edwards

**Affiliations:** University of Dundee, Dundee, United Kingdom

## Abstract

**Context** The oral cavity hosts the second most diverse microbiome in the human body; a complex ecosystem interacting with the host across multiple distinct ecological niches. Advances in sequencing abilities have transformed our understanding of the oral microbiome, but significant conceptual and methodological challenges remain.

**Objectives** This review explores the terminology that underpins microbiome research highlighting ambiguity and heterogeneity among current research and the challenges that arise when translating oral microbiome research to clinical dentistry.

**Current knowledge** While associations between oral microorganisms, oral microbial communities and both oral and systemic disease are increasing, current microbiome analyses alone cannot yet account for a number of important factors including host response, functional variation and strain level differences that determine pathogenic potential.

**Conclusion** A future with the ability to use microbiome analyses for diagnostic, prognostic and therapeutic purposes requires careful hypothesis-driven research with a combined multi-omics approach.

## Key points

- Current microbiome research provides insights into the composition of the oral microbiome but does not explain important functional changes.
- The oral microbiome is highly resistant to change, making therapeutic modulation challenging.
- Ongoing research exploring functional deviations will be key to improving our ability to assess individual disease risk.

## Introduction

The oral cavity has several distinct and unique interconnecting environments, supporting a range of diverse microorganisms. The complexity of the mouth results in the second most diverse microbiome in the human body, second to that of the gastrointestinal tract. This diversity can be explained by the variety of surfaces that range from the hard tissues of the teeth to soft tissues sites including the tongue and both keratinised and non-keratinised epithelia. Habitats also vary in the availability of oxygen, dominant sources of nutrition and the exposure to external factors including oral hygiene measures, eating, drinking and smoking.

Even in generally healthy individuals the oral microbiome can have an impact far beyond the oral cavity. Established links between oral microorganisms and extra-oral disease include the role of oral *Streptococci* in infective endocarditis,^1^ the association of *Porphyromonas gingivalis* and its virulence proteins with the brain of patients diagnosed with Alzheimer's disease, the presence of oral microorganism in the joints of patients diagnosed with rheumatoid arthritis and infections of the placenta that result in adverse pregnancy outcomes.^2^ Therefore, it is not surprising that the range of locations and number of pathologies associated with oral bacteria has expanded as our knowledge of the human microbiome has advanced.

This paper aims to define what the oral microbiome is, its limitations and what aspects of our understanding of the oral microbiome influence the dental management of patients.

## The advancement of computational power has accelerated microbiome research

Next generation sequencing platforms, including Illumina, Oxford Nanopore Technologies and Pacific Biosciences, advancements in computational power and artificial intelligence have accelerated research into microbiomes in recent years, so much so that an extension to the range of SI prefixes was required in 2022, including quetta, ronto and ronna, to accommodate for values in data science exceeding the previous range (i.e., kilo, mega, giga).^3^ To understand the vast volumes of data arising from microbiome research, it is important to have clearly defined terms, something that has been highlighted as a deficiency in this research area.^4^ Table 1 defines some commonly used terms in microbiome research, and these definitions alone can already give an indication of the challenges that arise when interpreting microbiome research. Much microbiome research for example is limited to metataxonomic evaluation of bacteria (analysis and taxonomic classification of 16S ribosomal ribonucleic acid [rRNA] regions) rather than whole microbiome analysis including all elements of the microbiome. For dental researchers and clinicians, understanding these distinctions is critical when interpreting microbiome-based research.

**Table 1:** Terms and definitions

**Term**	**Definition**
Microbiome	A characteristic microbial community in a reasonably well-defined habitat which has distinct physio-chemical properties as their theatre of activity^48^ (structural elements, metabolites, signal molecule and surrounding environmental conditions). Includes mobile genetic elements, e.g., phage, plasmids, prions, viroids, free DNA^45^
Microbiota	All living members forming the microbiome.^45^ Does not include mobile genetic elements
Metagenome	The collection of genomes and genes from the members of a microbiota. Obtained through metagenomics^49^
Metagenomics	Shotgun sequencing (random sequencing of fragments of DNA) of DNA extracted from a whole sample^49^ and subsequent analysis of reads
Metataxonomics	The process used to characterise the entire microbiome and create a metataxonomic tree. Requires amplification and sequencing of taxonomic marker genes,^49^ otherwise known as amplicon sequencing. Usually involves sequencing of regions of the 16S rRNA gene
Dysbiosis	Imbalances in the composition of bacterial microbiota,^50^ relying on the knowledge of what is a eubiotic system.^51^ Or a compositional and functional alteration in the microbiota that is driven by environmental and host factors that perturb the ecosystem to an extent that exceeds its resistance and resilience capabilities^16^
Eubiosis	Balance of the microbiota community that when disturbed becomes dysbiosis.^52^ A ‘healthy' community
Pathobiont	A symbiont that has the potential to cause pathology under certain conditions^25^
Pathogen	A microorganism that causes or can cause disease. A microbe that can cause damage in a host^53^
DNA, deoxyribonucleic acid; rRNA, ribosomal ribonucleic acid	

## Practical challenges associated with oral microbiome research

A critical step in analysis of the microbiome is acquisition of deoxyribonucleic acid (DNA) from an appropriate sample. It can be difficult to extract an adequate mass of DNA from samples obtained from the oral cavity (including saliva, swabs and plaque).^5^^,^^6^^,^^7^ Frequently the quantity of DNA extracted is sufficient only for amplicon sequencing, usually of the 16S rRNA gene. This is because amplicon sequencing works by amplification of target sequences, enabling the analysis of these selected sequences from a very low starting mass. Because shotgun metagenomic sequencing does not involve amplification, it requires a higher starting mass of DNA for analysis, however, it has a clear advantage in enabling the potential analysis of all DNA within a sample, not just selected small amplicons. Meaningful shotgun metagenomic sequencing is difficult because the low biomass samples also introduce a higher risk of issues where any contaminant DNA (from reagents or the environment) can significantly skew findings.^8^ This is because, while contamination is also a consideration in amplicon sequencing, any contaminant DNA is amplified alongside the target sample DNA, so a low abundance of contaminant DNA will result in a low abundance of contaminant amplicons. However, in shotgun metagenomics, where there is no amplification, particularly where there is very low starting sample DNA, the presence of contaminant DNA will be sequenced alongside the target DNA and can substantially affect the proportion of true microbial reads compared to contaminant reads. Methods for increasing biomass, such as a period without routine toothbrushing^9^^,^^10^^,^^11^ for these low biomass samples can be used to increase yield, but with the possibility to introduce biases, by not providing a true representation of the participant‘s routine microbiome. Even if an appropriate amount of DNA is acquired, garnering representative data from high complexity samples such as from the oral cavity and other diverse microbiomes, is extremely computationally intensive.^12^

We therefore encounter our first issue, which is that 16S rRNA amplicon sequencing remains the most accessible methodology for a number of reasons:

- Lower sequencing cost
- Lower computational burden
- Simpler downstream analysis
- Can be used for low biomass samples
- Fits existing workflows.

However, 16S rRNA amplicon sequencing has challenges surrounding interpretation of results, where methodological limitations can affect both technical reproducibility and taxonomic classification. For example, variability in results can be observed in different sequencing runs from DNA obtained from the same sample.^13^ Further, taxonomic assignment relies on pre-existing reference databases, meaning that classification accuracy is constrained by database completeness and curation. Additionally, the 16S rRNA gene is present in varying copy numbers in different species, some with identical copies and some with intragenomic heterogeneity^14^ leading to significant challenges when attempting to calculate accurate abundance data^15^ that is often used to characterise microbial shifts in dysbiosis.

## What is oral dysbiosis

We will now consider the challenge relating to defining dysbiosis. The words we use to describe bacterial communities of the microbiome are not as straightforward as ‘healthy' and ‘dysbiotic'. The simple definition of dysbiosis is the imbalance of the bacterial microbiota, a change from a balanced community,^16^ but this definition requires a clear understanding of what constitutes a balanced or ‘eubiotic' microbiome.^17^ This is because health or dysbiosis in this definition can involve the same taxa with varying abundance. Various investigations into what constitutes a core microbiota generally support the existence of a common set of species in a healthy mouth, but the variation in abundance of these species is large and not insignificant.^18^ Other research has identified geographical variation in the core oral microbiota with significant variation in abundance of core species which cannot be directly linked with any clear factor.^19^

The main deficiencies in the use of the term dysbiosis for the oral microbiome are that, while it can describe changes in the composition of the microbial communities that are associated with health and disease, it does not identify functional differences in those communities, nor does it identify the role of the host response. Thus alone, it cannot be used to determine disease risk for supposed dysbiotic conditions including periodontitis and caries.

## Pathogens, pathobionts, the host response

Just as the definitions of dysbiosis, microbiome and health are not as they seem, genomics and metagenomics have muddied the waters in whether something can truly be classed as a pathogen or not. Koch's postulates^20^ were the first criteria aimed at defining the role of pathogens in infection, identifying a single species with a role in causing infection, however, it was Koch's team themselves who recognised that the four postulates do not in fact work for every infection, particularly in the instance of being an asymptomatic carrier of a proposed pathogen and the inability to culture that which is not alive (e.g., viruses). Centuries on from this, we still encounter the debate of when is a pathogen a pathogen, and caries and periodontitis are clear examples demonstrating this confusion, where we don't have a single pathogen causing a disease, and where we have pathogens existing as normal constituents of a healthy community. While there is clearly evidence that certain organisms cause disease, and molecular biology can demonstrate that specific virulence proteins induce host damage, genomics has taught us that supposedly pathogenic species with pathogenic virulence genes can also exist in a state of passivity, without expressing virulence factors and without causing disease. What is clear now is microbial roles are dependent on their context; some commensals may exhibit pathogenic behaviour under specific host conditions and vice versa.

This has given rise to another term of ‘pathobiont', an opportunistic organism which exists in the normal, healthy microbial community which is able to promote pathology under certain conditions. *P. gingivalis* for example, the typical periodontal ‘pathogen' which exists in the ‘core microbiota', but under some conditions considered to be ‘dysbiotic' can cause periodontitis, while when in a ‘eubiotic' community, it seemingly does not induce the host response for tissue destruction.^21^^,^^22^ In a similar vein, but altogether different disease process, *Candida albicans* in most people would be considered a commensal, in some it causes localised candidiasis and in severely immunocompromised patients it has the potential to cause life threatening invasive candidiasis.^23^ *Fusobacterium nucleatum* is another species that is commonly identified in both health and disease, but is being increasingly implicated in disease both in the mouth and beyond.^24^ It may, at this point come as no surprise that the definition of term ‘pathobiont' is also being debated even in its young age,^25^ again because of the lack of clarity in what it really means and the reliance on understanding the meaning of dysbiosis compared to healthy microbial communities.

This brings us to something that we have long known in periodontology: the importance of the host response in relation to the development and progression of diseases associated with the microbiome. While the periodontal pocket harbours a distinct microbial community^26^ and changes in abundance are associated with disease severity,^27^^,^^28^^,^^29^ pathogenic species are not exclusive to diseased sites and health-associated species are often still present in diseased sites.^28^^,^^30^ These observations indicate that although microbial shifts are a cornerstone of periodontitis aetiology, testing for specific microbial species or microbial community changes cannot currently reliably determine disease severity in the context that the host response plays a significant role in driving tissue destruction.

With the current evidence and unclear understanding of the constituents of a ‘healthy' oral microbiome, as well as challenges in obtaining accurate values of absolute or differential abundance^31^ it is difficult use microbiome data alone to determine whether deviations from ‘normal' represent a clinically relevant risk. Additionally, while some elements of microbiome analysis can give some indication of the pathogenic potential of a microbiome, it cannot provide clinically useful information for diagnosis, staging or predicting disease progression at this stage, because it cannot capture the individual host response that ultimately governs the severity of tissue damage. Clinically, this underscores that oral microbiome findings should be interpreted as adjunctive and contextual information, rather than standalone diagnostic or prognostic markers. Their relevance lies in informing understanding of disease mechanisms and risk interpretation rather than immediate clinical decision-making.

## The stability of the oral microbiome and its influences

The ‘healthy' oral microbiome when evaluated metataxonomically is very stable over time within the same individual.^32^^,^^33^ This stability is largely driven by factors such as biofilm formation and the presence of saliva, which contribute to resilience against change.^34^ The dental pellicle has a range of mechanisms through which the oral microbiota can adhere to both hard and soft tissue surfaces and initiate biofilm formation.^35^ The rapid establishment and re-establishment of this pellicle and the presence of bacteria in protected niches results in an impossible task of completely eradicating all oral microbiota. This biofilm-related resilience also contributes to why the oral cavity is so quickly recolonised following oral hygiene methods or professional mechanical plaque removal; while much of the biofilm might be removed, millions of bacteria ultimately remain, allowing the re-establishment of the early biofilm.

The periodontal pocket is further resistant to change because of its environmental niche that is quite distinct from the rest of the mouth leading to a resilient diseased community.^34^ It results in an anaerobic environment protected from mechanical forces such as eating and brushing allowing a microbial community that differs substantially to that associated with health.

In contrast with the gut, where we can, for example, change the microbiome through interventions such as faecal microbiome transplantation,^36^ the oral cavity is much less receptive to external change from, for example, probiotics^37^ or microbiota transplants. Consequently, one key aspect in research in oral microbiota transplantation focuses on strategies to provide sustained contact of transplanted microbiota to the mouth using hydrogels and intra-oral trays,^38^ to try and improve colonisation.

**Fig. 1:**
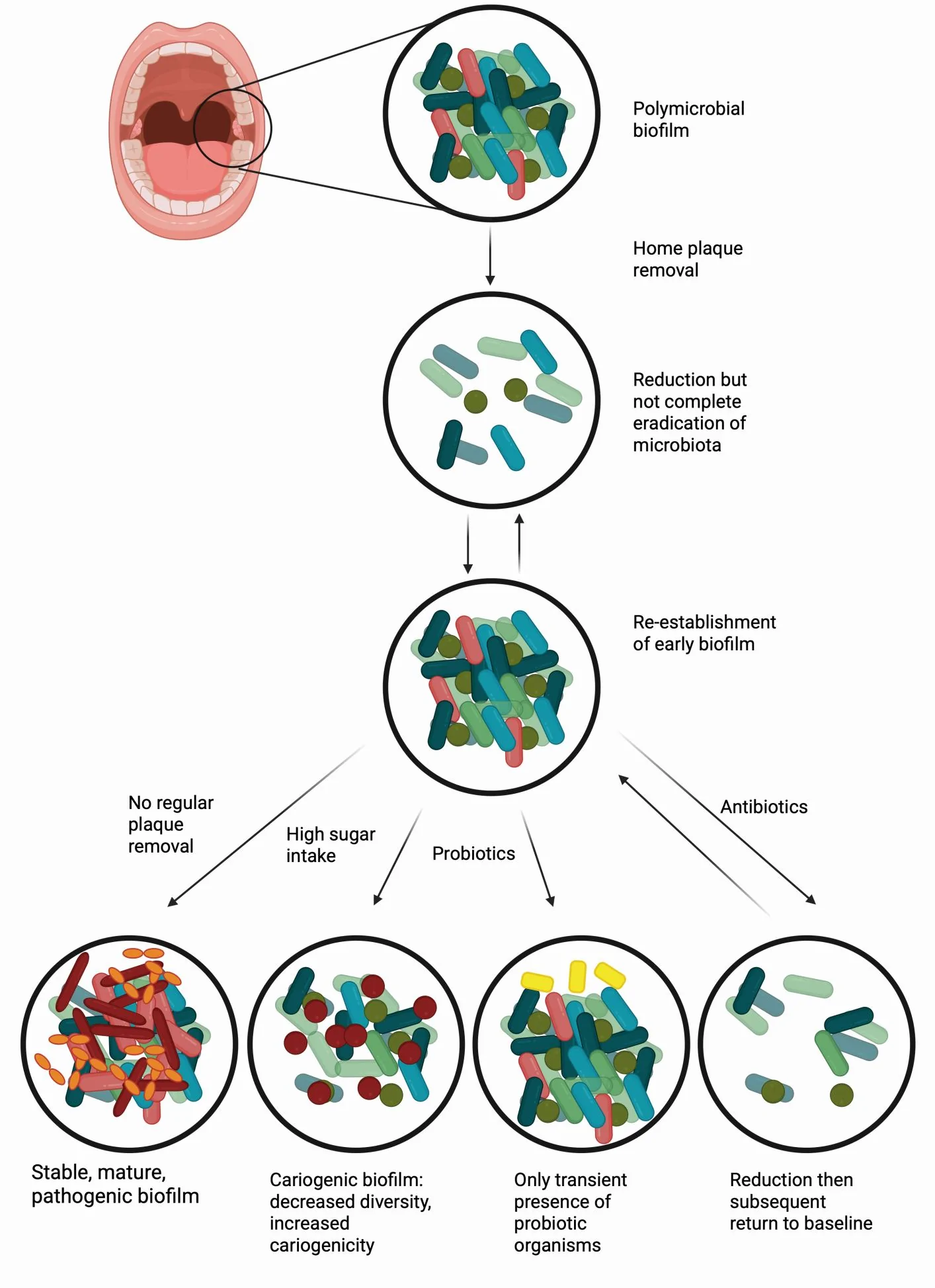
The stability of the oral microbiome. Created with BioRender.com

Figure 1 illustrates further why, despite external interventions to remove or add to the oral microbial community, the oral microbiota is resistant to change from short-term interventions. It highlights how regular mechanical plaque removal reduces microbial load, but does not fully eradicate the community, allowing early biofilm re-establishment. In contrast, a lack of regular plaque control promotes a change to a stable, mature, pathogenic biofilm, while dietary factors such as high sugar intake select for a less diverse, cariogenic community. Transient interventions such as probiotics only have temporary effects on biofilm composition.

Clearly, the oral microbiome has unique features giving rise to resilience to external factors, making regular mechanical plaque removal and effective preventive care the most predictable method in shifting from a diseased to healthy state. This resilience additionally highlights a key reason why gut microbiome research does not readily translate to the oral microbiome.

## What can current oral microbiome data tell us about systemic disease risk?

There is increasing evidence linking oral microorganisms with systemic disease. These include links between *P. gingivalis* and Alzheimer's disease,^39^ *F. nucleatum* and colorectal cancer^40^ and various oral organisms that have been identified in the bloodstream as bacteraemia.^41^

While the oral microbiome has the potential to give valuable insights into human health, it cannot currently be used to reliably predict individual risk for extra-oral disease associated with oral microorganisms. Metagenomic and metataxonomic analyses often lack the resolution needed to distinguish strain-level differences, which can be a defining factor in the pathogenic potential of an organism. As previously described, these approaches have additional challenges relating to the accurate reporting of changes in microbial abundances or measures of absolute abundance, making it difficult to assess the degree of ‘dysbiosis'.^42^ Additionally, because dysbiosis is not solely defined by the compositional changes, but also by functional changes, measuring compositional changes alone cannot capture the full picture of disease risk.^43^

While general profiling can identify patterns at a population level, it lacks the specificity, context and mechanistic understanding necessary for personalised risk assessment. It would be possible to develop specific tests with specific microbial and immune markers; these would no longer be considered microbiome testing, but rather precise diagnostics based on validated biomarkers.

## Conclusion

We cannot assume that an individual species or strain is inherently pathogenic nor can we define what dysbiosis is in the absence of a more complete understanding of eubiosis. Similarly, we cannot assume that different degrees of dysbiosis are always associated with degrees of pathosis, nor is there currently an established framework for measuring degrees of dysbiosis from the norm. Instead, we can consider the pathogenic potential^44^ an individual species or community shift has, which requires specific questions that metagenomics and metataxonomics can't answer alone.

While microbiome research has yielded valuable information about which organisms are present, comprehensive understanding requires multiple approaches including microscopy, culturing, metagenomics, metatranscriptomics, metaproteomics and metabolomics to reveal both composition and function.^45^

Thus, despite the ease of accessibility of some aspects of -omics research, we cannot fully understand the relationships and impacts of the microbiota in an environment without knowing what an individual species is capable of in the first place. As Kumar *et al.* have detailed, current sequencing methodologies have transformed our understanding of the oral microbiome, while identifying technical and interpretive limitations.^46^ Similarly, Overmann *et al.* emphasise the difficulties or impossibilities in -omics research when encountering not-yet-cultured microorganisms,^47^ and much of these issues can also be related to poorly characterised and under-researched organisms such as *F. nucleatum* and the Fusobacteria genus as a whole. Because current algorithms rely on existing databases, incomplete genomic annotation continues to challenge our understanding of functional variation.

While oral-systemic associations are strong at the population level, individual, current diagnostic tests based on bacterial composition should be interpreted with caution because they cannot account for differences in microbial gene expression or host response.

Understanding the roles of individual species or specific changes to the microbiome requires specific questions to be asked. Meaningful insights into microbiome-associated health and disease require targeted, hypothesis-driven studies that connect microbial identity with function and host response.

For dental practice, oral microbiome findings provide contextual insights into disease risk, often on a population level and in varying levels of detail and clinical significance. They add value to our overall understanding, support informed discussions with patients and guide future directions in research but should not be used to guide individual diagnosis or treatment decisions alone. Established preventive and therapeutic strategies remain paramount.

## Data Availability

No datasets were generated or analysed for this review.
